# Higher Circulating Cortisol in the Follicular vs. Luteal Phase of the Menstrual Cycle: A Meta-Analysis

**DOI:** 10.3389/fendo.2020.00311

**Published:** 2020-06-02

**Authors:** Ajna Hamidovic, Kristina Karapetyan, Fadila Serdarevic, So Hee Choi, Tory Eisenlohr-Moul, Graziano Pinna

**Affiliations:** ^1^Department of Pharmacy Practice, College of Pharmacy, University of Illinois at Chicago, Chicago, IL, United States; ^2^Department of Epidemiology, Erasmus Medical Centre Rotterdam, Rotterdam, Netherlands; ^3^Department of Psychiatry, College of Medicine, University of Illinois at Chicago, Chicago, IL, United States; ^4^The Psychiatric Institute, Department of Psychiatry, College of Medicine, University of Illinois at Chicago, Chicago, IL, United States

**Keywords:** cortisol, hypothalamic-pituitary-gonadal (HPG) axis, hypothalamic-pituitary-adrenal (HPA) axis, menstrual cycle, follicular, luteal

## Abstract

Although results of animal research show that interactions between stress and sex hormones are implicated in the development of affective disorders in women, translation of these findings to patients has been scarce. As a basic step toward advancing this field of research, we analyzed findings of studies which reported circulating cortisol levels in healthy women in the follicular *vs*. luteal phase of the menstrual cycle. We deemed this analysis critical not only to advance our understanding of basic physiology, but also as an important contrast to the findings of future studies evaluating stress and sex hormones in women with affective disorders. We hypothesized that cortisol levels would be lower in the follicular phase based on the proposition that changes in levels of potent GABAergic neurosteroids, including allopregnanolone, during the menstrual cycle dynamically change in the opposite direction relative to cortisol levels. Implementing strict inclusion criteria, we compiled results of high-quality studies involving 778 study participants to derive a standardized mean difference between circulating cortisol levels in the follicular vs. luteal phase of the menstrual cycle. In line with our hypothesis, our meta-analysis found that women in the follicular phase had higher cortisol levels than women in the luteal phase, with an overall Hedges' *g* of 0.13 (*p* < 0.01) for the random effects model. No significant between-study difference was detected, with the level of heterogeneity in the small range. Furthermore, there was no evidence of publication bias. As cortisol regulation is a delicate process, we review some of the basic mechanisms by which progesterone, its potent metabolites, and estradiol regulate cortisol output and circulation to contribute to the net effect of higher cortisol in the follicular phase.

## Introduction

Women exhibit high prevalence of stress-related disorders, such as major depressive disorder (MDD) and anxiety spectrum disorder (1–9). Importantly, the increase in prevalence of these disorders is observed during periods of drastic hormonal changes, such as puberty, the pre-menstrual period, pregnancy, postpartum and menopause (10–12). These observations suggest that interactions between sex hormones, regulated by the hypothalamic-pituitary-gonadal (HPG) axis, and cortisol, a stress hormone under the control of the hypothalamic-pituitary-adrenal (HPA) axis, may be critical determinants of stress-related disorder development and progression.

Research evaluating stress effects in MDD and anxiety disorders demonstrates a blunted cortisol response to psychosocial stress in female patients compared to their respective controls [for a meta-analysis, see (13)]. However, although these research studies provide valuable information, they only examine function of the HPA axis, without evaluating how sex hormones influence it.

The number of studies evaluating interactions between the provoked HPA and the HPG axes is limited in both diseased as well as healthy participants. Results of studies comparing reactivity to psychosocial stress in healthy women suggest that cortisol output is higher in the luteal *vs*. follicular phase of the menstrual cycle (14, 15). However, their small sample size and opposite findings from other studies (16–19) indicate that more research needs to be completed before a conclusion can be drawn. Additional studies, implementing strict verification of menstrual cycle phase, stress manipulation and participants' healthy status, are needed.

An even more fundamental question, though, is related to the physiological relationship between the HPA and HPG axes under unprovoked, tonic conditions. However, results from human laboratory and observational studies in healthy volunteers evaluating basal cortisol levels across the menstrual cycle range broadly. Thus, we focused on the function of the HPA axis with the hypothesis that there would be a higher physiological output of cortisol during the follicular compared to the luteal phase of the menstrual cycle. Our hypothesis was based on the finding that the progesterone metabolite allopregnanolone positively modulates gamma-aminobutyric acid (GABA)_A_ receptors via an allosteric binding site to potentiate inhibitory signaling (20) and enhance the negative feedback on the HPA axis (21, 22). Therefore, during the luteal phase, when allopregnanolone levels are high, cortisol levels would be expected to decrease relative to the follicular phase, when allopregnanolone levels are low.

Tonic levels of cortisol across the menstrual cycle have been reported as higher for example, (23, 24) or unchanged (25, 26) in the follicular vs. luteal phase. These discrepancies are rooted in marked methodological differences across studies. Hence, our analysis only included high-quality research studies implementing strict criteria and phase identification. Based on mechanistic considerations of basic research studies (reviewed in the Discussion section) that have reported the effects of neuroactive steroids on the HPA axis function, we predicted a surge in cortisol during the early/mid-follicular phase. Our meta-analysis, indeed, shows that circulating cortisol levels change dynamically as a function of menstrual cycle phase, suggesting cortisol is specifically required during the early/mid follicular phase to mediate adaptive physiological processes in response to environmental stimuli, when both estradiol and progesterone are low.

## Methods

### Search Strategy

We conducted a literature search in PubMed, Web of Knowledge and PsychInfo, and included eligible studies published through December 5th, 2019. Two authors (AH and KK) completed their search independently according to the Preferred Reporting Items for Systematic Reviews and Meta-Analyses (PRISMA) guidelines (27). Any discrepancies were reconciled by reviewing the literature jointly for specific points of difference. We used the following search string: [(“Cortisol”) AND (“Menstrual” OR “Luteal” OR “Follicular”) for (DOCUMENT TYPE: (Article); LANGUAGE: English; SUBJECTS: Human)]. We compiled the results in EndNote X8.

### Inclusion/Exclusion Criteria

This meta-analysis evaluated tonic peripheral cortisol levels of healthy menstruating female study participants across follicular vs. luteal phases of the menstrual cycle. Studies were considered eligible if a baseline value was provided prior to a laboratory intervention (for example, psychosocial stress procedure or exercise), if samples were collected longitudinally in a naturalistic (or a laboratory) setting across the menstrual cycle, if an experimental design evaluating a disease state included a healthy control or if an intervention included a placebo control. The exclusionary criteria implementation was carried out in a two-step approach.

In the first step, study abstracts (*N* = 2,225) were excluded if they were: (1) abstracts, review papers or case studies, (2) animal studies, or evaluation of cell lines, (3) male-only evaluations, (4) abstracts which only mentioned one menstrual phase (luteal or follicular) as a means of controlling for menstrual cycle phase (i.e., not as a comparison of the two phases), (5) studies evaluating a diseased population (including smokers or other substance use disorder population) or implementing a menstrual phase-specific intervention (without a placebo control). This category also included abstracts describing pregnant as well as women in the peri- or post-menstrual phases, as well as women who were on oral contraceptives. Finally, abstracts describing athletes or women who experienced early life trauma were also coded in this category. The remaining abstracts were excluded if they: (6) did not mention cortisol (blood, salivary or urinary), (7) were overlapping study participants with an already published study, and (8) if they described a procedure (such as IV fertilization, for example) which could cause changes in circulating cortisol due to anticipation.

In the second round of exclusion criteria implementation, we evaluated full articles. We excluded papers which did not evaluate groups according to menstrual cycle phases, measure cortisol, have a healthy, non-athletic control group, or report mean and/or variance and were published more than 25 years ago. Furthermore, we excluded papers which re-administered stress (as this can distort baseline cortisol levels), performed non-linear cortisol modeling, were non-original, written in languages other than English, or had overlapping participants. Given the rapid decline of cortisol in the morning, studies which collected cortisol during morning times that varied ≥2 h or did not mention what time of the day cortisol sample collection -took place were excluded, as well as studies which determined cycle phase using self-report.

### Data Extraction

Information on the following variables was collected: (1) age, (2) BMI, (3) day of follicular and luteal phase of cortisol collection, (4) phase estimation method, (5) time of day of cortisol collection, and (6) physiological source of cortisol level. Regarding the length of phase variable, whereas some studies reported a range of days, others reported multiple, exact days of the cycle on which cortisol was reported. In the event that a study reported cortisol values across multiple days of the menstrual cycle, data from the day closest to the beginning of the cycle (day 1) for follicular phase, and day 21 of the luteal phase were extracted to reflect the greatest contrast of estradiol and progesterone levels across the cycle. In the event that cortisol was reported on multiple “sub-phases” (for example, early-follicular, mid-follicular), again, data from the day closest to the beginning of the cycle (day 1) for follicular phase, and day 21 of the luteal phase were extracted. If cortisol was collected across multiple menstrual cycles (for example, two menstrual cycles), values from the last cycle were reported. In the event that studies reported cortisol values at multiple times of the day (for example, morning and evening), or multiple sources (for example, salivary and plasma), the most frequently reported time (morning) and source (blood) in the remaining studies were used to extract cortisol values.

### Data Analysis

Analyses of were carried out by first calculating the Cohen's *d* effect size (28). In the event mean and variance values were provided for sub-groups of women in follicular and luteal phases, those were combined using the following formulas:

$$\begin{array}{r} M=\frac{N_{1}M_{1}+N_{2}M_{2}}{N_{1}+N_{2}}\\ SD=\\ \sqrt{\frac{\left(N_{1}-1)\;SD_{1}^{\;2}\text{​}+(N_{2}\text{​}-1)\;SD_{2}^{\;2}\text{​}+\frac{N_{1}N_{2}}{N_{1}+\;N_{2}}\text{ ​}(M_{1}^{\;2}\text{​}+M_{2}^{\;2}\text{​}-\text{​ }2M_{1}M_{2}\right)}{N_{1}+N_{2}-1}} \end{array}$$

where, N_1_ = sample size group 1, N_2_ = sample size group 2, M_1_ = mean group 1, M_2_ = mean group 2, SD_1_ = standard deviation group 1, SD_2_ = standard deviation group 2.

We divided the mean difference between the two groups by the pooled standard deviation. Next, we used the J-correction factor to obtain the Hedges' *g* effect size, which corrects for small samples (29) and is considered small, medium, and large for values 0.2, 0.5, and 0.8, respectively. Within- and between-subject study designs were combined as described in Morris et al. (30). We used a random effects model to calculate the pooled effect size with an associated 95% CI and a *p*-value (31). We assessed source-study heterogeneity using the χ^2^-based Q test with its associated *p*-value. A statistically significant Q statistic suggests different effect sizes across studies, implying that methodological or population sample differences may be introducing variance across individual studies. We quantified heterogeneity using *I*^2^ with values 25, 50, and 75% suggestive of small, medium and large heterogeneity and calculated potential publication bias using the Classical Tests (32). We completed sub-analyses according to source (saliva vs. plasma) and time of day (morning vs. afternoon). The meta-analysis was performed using the “escalc” function, and publication bias was assessed using the “ranktest” function in “metafor” package (33) in R.

## Results

### Characteristics of Individual Studies

**Figure 1:**
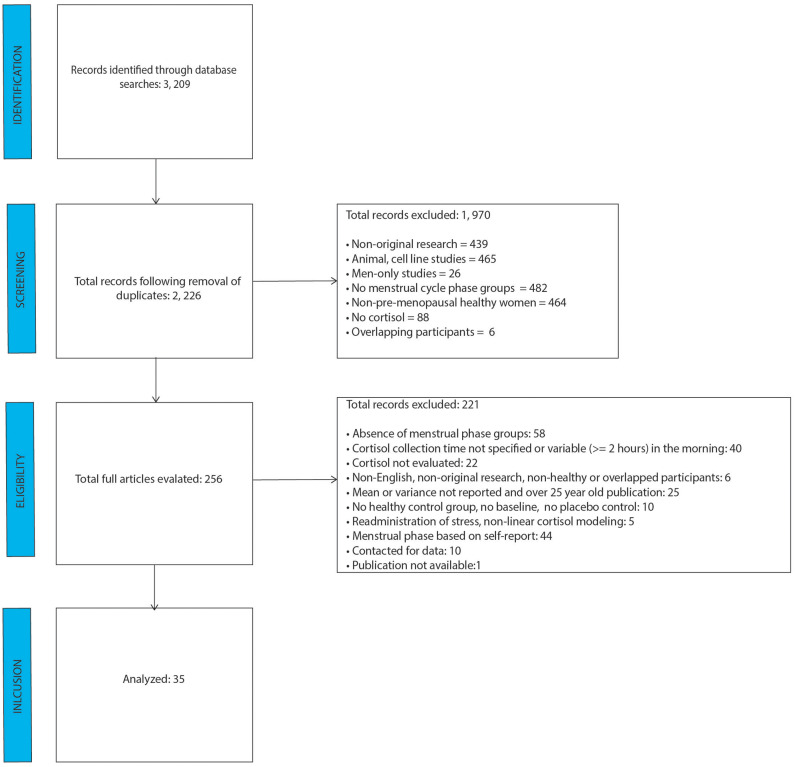
PRISMA flow diagram.

After removal of duplicate studies, literature search identified 2, 226 individual abstracts as shown in the PRISMA figure (Figure 1). Those abstracts were screened for relevance and coded for exclusion reasons. The greatest number of abstracts excluded was based on absence of menstrual cycle groups, followed by cell line/animal research studies. A total of 256 full-text articles were reviewed for relevance, of which 221 were excluded. Of the 221 studies, 44 were excluded based on the self-report nature of menstrual cycle phase determination, and 40 were excluded because they either didn't mention cortisol collection time, or the morning sample was collected at times which varied by two or more hours. The analysis included data from 35 final studies.

As shown in Table 1, most of the studies included participants in their 20s, with a BMI below 25. Whereas, some studies incorporated fine-grained sub-phases of follicular and luteal phases (see Table 2), others defined the phases as day 1–14 and 15–28. Phase estimation was determined via progesterone level acquisition, LH surge measurement, basal body temperature measurement, or pelvic ultrasound. Times of cortisol collection were in the morning, afternoon/evening or over several hours, and the source was saliva, plasma or urine. Whereas, Supplementary Table 1 shows all the days/phases of menstrual cycle, time of collection and sources of cortisol across all the studies, Table 2 only shows the actual day (or range of days), time and source of cortisol which were included in the analysis. These two tables are provided in order to increase transparency of reporting, as several studies reported several values, for which we implemented the rules as specified in the Methods Section Data Extraction.

**Table 1:** Participant information from individual studies.

**References**	**Sample size (*****N***)^*^		**Age [mean (SD)]**	**BMI [mean (SD)]**
	**Follicular Phase**	**Luteal Phase**		
Andreano et al. (34)	20	24	___	___
Barbarino et al. (35)	5	6	___	___
Beck et al. (36)	20		___	___
Hoeger Bement et al. (37)	20		20.9 (1.0)	23.0
Bricout et al. (38)	11		25.5 (7.6)	19.9
Cannon et al. (39)	7	8	___	___
Carr et al. (40)	4		___	___
Caufriez et al. (41)	10		30.0	21.8 (0.9)
Childs et al. (42)	29	23	21.9 (0.8)	22.3 (0.3)
Collins et al. (43)	15		29.5	___
Espin et al. (14)	30	30	19.3 (1.7)	21.7 (4.1)
Genazzani et al. (44)	5		___	___
Heitkemper et al. (45)	25		33.1 (5.3)	23.6 (4.9)
Huang et al. (46)	18	18	22.0 (2.4)	20.0 (2.8)
Inoue et al. (26)	9		23.7 (5.6)	___
Judd et al. (47)	6	6	___	___
Kasa-Vubu et al. (48)	10	14	29.4 (8.5)	24.0 (4.3)
Kerdelhué et al. (23)	11		___	___
Kirschbaum et al. (17)	19	21	23.4 (3.3)	21.7 (2.4)
LeRoux et al. (49)	9	9	21.8 (2.3)	22.5 (2.4)
Liu et al. (50)	6		___	___
Lombardi et al. (51)	20		26.2	___
Maki et al. (52)	20	20	27.0 (5.6)	25.0 (5.0)
Ohara et al. (53)	7		22.3 (1.0)	20.5 (2.1)
Paoletti et al. (54)	14		31.5 (2.7)	24.2 (2.0)
Parry et al. (55)	30		37.2 (5.8)	___
Rasgon et al. (56)	5		27.0 (4.0)	___
Reynolds et al. (57)	61		21.7 (3.4)	___
Roche and King (58)	23	23	24.2 (3.9)	23.6 (3.8)
Stewart et al. (59)	4		24.6 (4.5)	24.7 (2.1)
Su et al. (60)	10		30.8 (4.9)	___
Timon et al. (25)	20		___	21.3 (2.1)
Tulenheimo et al. (61)	14		___	___
Villada et al. (62)	13	17	19.0 (1.5)	21.3 (4.0)
Wolfram et al. (63)	29		26.3 (3.9)	22.1 (2.9)

* *Only one sample size (for follicular and luteal phases) is listed for within subject design studies. The total sample size is 778*.

**Table 2:** Menstrual cycle and outcome measure information from individual studies.

**References**	**Menstrual cycle**			**Cortisol**	
	**Follicular phase**	**Luteal phase**	**Phase estimation**	**Time**	**Source**
Andreano et al. (34)	1–7	18–24	Estradiol and progesterone	Afternoon	Saliva
Barbarino et al. (35)	4–8	20–24	Estradiol and progesterone	Morning	Plasma
Beck et al. (36)	10	24	LH surge	Morning	Plasma
Hoeger Bement et al. (37)	“Mid-follicular”	“Mid-luteal”	LH surge	Afternoon	Saliva
Bricout et al. (38)	“Mid-follicular”	“Mid-luteal”	Estradiol and progesterone	24-h	Urine
Cannon et al. (39)	1–14	15–28	Progesterone	24-h	Urine
Carr et al. (40)	1	21	LH surge	Morning	Plasma
Caufriez et al. (41)	3–8	23–28	Basal body temperature	24-h	Urine
Childs et al. (42)	3–10	16–24	LH ovulation test	Morning	Plasma
Collins et al. (43)	5–7	22–25	Basal body temperature	Morning	Plasma
Espin et al. (14)	5–8	20–24	Basal body temperature	Afternoon	Saliva
Genazzani et al. (44)	1	21	LH surge	Morning	Plasma
Heitkemper et al. (45)	1	22	LH ovulation test	Morning	Urine
Huang et al. (46)	1–4	24–28	Estradiol and progesterone	Afternoon	Saliva
Inoue et al. (26)	1–14	21–28	Estradiol and progesterone	Morning	Plasma
Judd et al. (47)	3–5	20–24	LH ovulation test	10-h	Serum
Kasa-Vubu et al. (48)	1–14	15–28	LH and progesterone	24-h	Plasma
Kerdelhué et al. (23)	1	21	LH surge	Morning	Serum
Kirschbaum et al. (17)	4–7	21–25	Estradiol and progesterone	Afternoon	Plasma
LeRoux et al. (49)	8–10	20–22	Estradiol and progesterone	Morning	Saliva
Liu et al. (50)	1–5	20–22	Pelvic Ultrasound	Morning	Plasma
Lombardi et al. (51)	5–7	22–26	LH surge and progesterone	Morning	Serum
Maki et al. (52)	2–4	22–24	LH ovulation test	Afternoon	Saliva
Ohara et al. (53)	1–14	15–28	LH ovulation test	Morning	Saliva
Paoletti et al. (54)	5–8	21–24	Basal body temperature	Morning	Serum
Parry et al. (55)	6–8	26–28	LH ovulation test	Morning	Plasma
Rasgon et al. (56)	2–9	7–14	LH ovulation test	Morning	Plasma
Reynolds et al. (57)	7–10	20–23	LH ovulation test	Afternoon	Saliva
Roche and King (58)	1–14	15to 28	Estradiol and progesterone	Morning	Plasma
Stewart et al. (59)	7	21	Progesterone	12-h	Plasma
Su et al. (60)	3–7 days after the end of menses	21	Progesterone	Morning	Plasma
Timon et al. (25)	1–2	21–22	Basal body temperature	Morning	Urine
Tulenheimo et al. (61)	6–9	21–24	Progesterone	Morning	Plasma
Villada et al. (62)	5–8	20–24	Basal body temperature	Afternoon	Saliva
Wolfram et al. (63)	2–6	21–24	LH ovulation test	CAR	Saliva

*CAR, Cortisol Awakening Response*.

### Evaluation of Standardized Mean Difference in Cortisol Levels Across Menstrual Cycle

**Figure 2:**
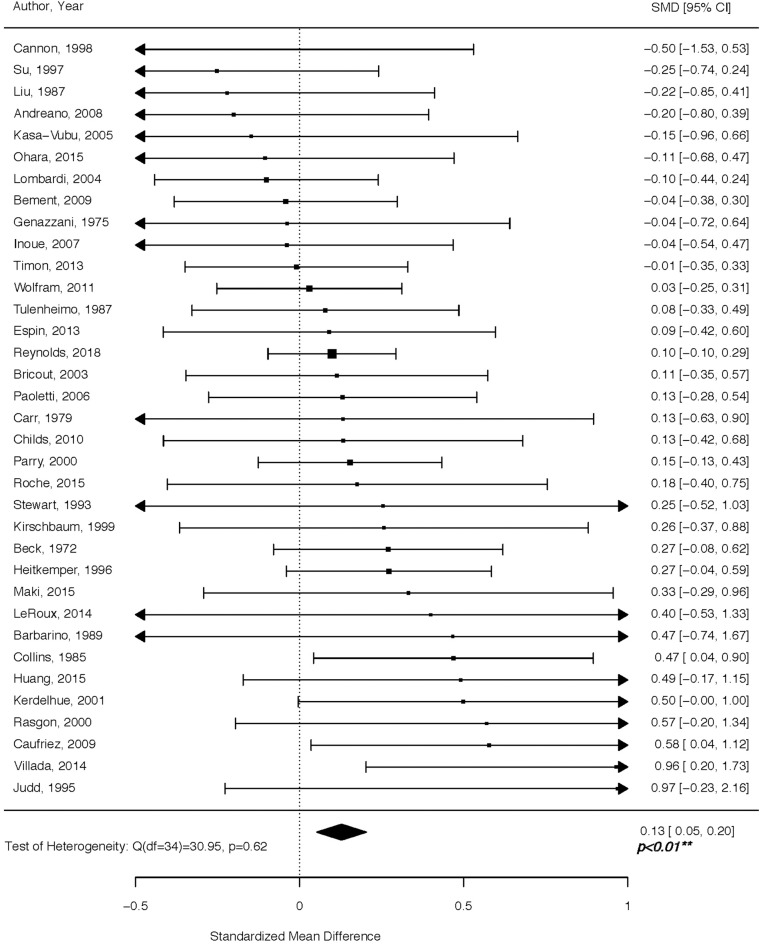
Forest plot of cortisol levels across the menstrual cycle. Positive standardized mean difference (SMD) means that cortisol levels were higher in the follicular vs. luteal phase.

Women in the follicular phase had higher cortisol levels than women in the luteal phase, with an overall Hedges' *g* of 0.13 (*p* < 0.01) for the random effects model (Figure 2). The confidence interval range was between 0.05 and 0.20. No significant between-study difference was detected (tau^2^ =0; H = 1.0), with the level of heterogeneity in the small range (*I*^2^ = 0%, Q = 30.69; *p* = 0.63). The Rank Correlation Test for Funnel Plot Asymmetry resulted in Kendall's tau = 0.14 (*p* = 0.23), indicating absence of small study effects.

### Sub-analysis of Cortisol Levels According to Source and Time of Day

Sub-analysis according to the biospecimen source showed a significant effect of plasma (*p* = 0.036) and marginally significant effect of saliva (*p* = 0.055). The time of day sub-analysis showed a significant effect of morning (*p* = 0.005), but not afternoon (*p* = 0.177). Table 3 displays all relevant sub-analyses statistics, including standardized mean difference, standard error, z and *p*-values, confidence intervals, degrees of freedom, Q statistic and its associated *p*-value.

**Table 3:** Results of sub-analyses according to the biospecimen source and time of day.

**Factor**	**SMD**						**Q**		
	**Value**	**SE**	**Z value**	***p*-value**	**CI.LB**	**CI.UB**	**df**	**Value**	***p*-value**
**Source**									
Plasma	0.12	0.0583	2.0907	0.0366	0.0076	0.2361	18	13.2214	0.7783
Saliva	0.1179	0.0615	1.9182	0.0551	−0.0026	0.2384	8	7.872	0.446
**Time of day**									
Morning	0.1363	0.0485	2.8088	0.005	0.0412	0.2314	21	16.6924	0.7296
Afternoon	0.0966	0.0716	1.348	0.1777	−0.0438	0.237	7	9.1638	0.2411

## Discussion

For decades, literature on cortisol has yielded mixed results with respect to its concentration in the follicular vs. luteal phase of the menstrual cycle. Implementing a comprehensive search of high-quality studies spanning a period of almost 50 years of research, we show that circulating cortisol levels are higher in the follicular vs. luteal phase. Cortisol regulation is a delicate process of extensive physiological processes working in concert to adjust responses to environmental stimuli. Below, we review mechanisms driving circulating cortisol levels to both increase and decrease across various menstrual cycle time-points, while noting that the net effect of these, or other, still unidentified processes, is a higher circulating cortisol during the follicular compared to the luteal phase, as reported in our analysis (Figure 2).

The paraventricular nucleus (PVN) of the hypothalamus integrates numerous circadian and environmental inputs to funnel information through neurons expressing corticotropin-releasing hormone (CRH). The release of CRH into the hypophyseal portal vasostructure enhances the synthesis and release of adrenocorticotropic hormone (ACTH) from the anterior pituitary, which, in turn, stimulates adrenal glands to synthesize and release cortisol to adapt metabolic processes and behavioral responses.

Intriguingly, PVN neurons express high levels of estrogen receptor beta (ER-β) and low levels of estrogen receptor alpha (ER-α) (64–66). Several research studies have demonstrated that estradiol, through its near equivalent affinity for the two estrogen receptor subtypes, can selectively decrease or increase HPA axis function. A high number of ER-β-expressing cells in the PVN are oxytocin and vasopressin immunoreactive (67–69), which complement the CRH neurons that also express ER-β (64). Stimulation of ER-β in the PVN results in a reduction of cortisol levels. In accord, both centrally- and peripherally-delivered ER-β selective agonists inhibit the HPA function (70). ER-α occupancy, on the other hand, has an indirect, trans-synaptic activation in the PVN. The peri-PVN region contains ER-α neurons, and their activation can impair glucocorticoid-mediated negative feedback regulation of the HPA axis (71). These opposing actions of estradiol—with ER-α amplifying, and ER-β reducing HPA function—are in agreement with laboratory observations that estradiol both enhances (72, 73) and inhibits (74, 75) HPA function. Hence, in the luteal phase, when estradiol levels are higher compared to the early/mid follicular phase, theoretically, depending on the extent of ER-β or ER-α expression and activation in or near the PVN, estradiol can either decrease or increase circulating cortisol levels.

**Figure 3:**
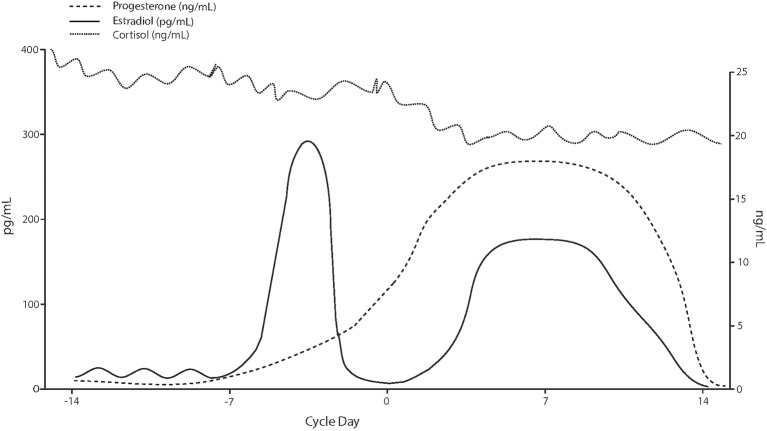
Hypothetical cortisol levels across the menstrual cycle based on values reported in the analyzed studies.

The activity of CRH neurons in the PVN is tightly regulated by inhibitory GABAergic interneuron populations (76). Allopregnanolone, a progesterone derivative resulting from conversion by 5α-reductase type I and 3α-hydroxysteroid dehydrogenase, is an endogenous neurosteroid and a potent, positive, allosteric modulator of the action of the inhibitory neurotransmitter GABA at GABA_A_ receptor. Studies in rodents show an inhibitory effect of allopregnanolone on the function of the HPA axis (77–79). This effect of allopregnanolone seems to be exerted through its action at GABA_A_ receptors, and subsequent inhibition on PVN neurons (80) under both basal and stressful conditions (81). In support of these findings, in addition to allopregnanolone, another potent GABA_A_ receptor modulator and deoxycorticosterone-derived steroid, tetrahydrodeoxycorticosterone (TH-DOC), also attenuates the HPA axis function (77, 82, 83). Therefore, in the luteal phase, under the physiological milieu of higher circulating progesterone levels and, most importantly, of its potent GABA_A_ receptor-modulating metabolite, allopregnanolone, lower circulating cortisol levels can be expected relative to the follicular phase. Not surprisingly, our meta-analysis has confirmed this expectation (Figure 3).

Once released in the blood flow, ~80% of circulatory cortisol is bound to corticosteroid-binding globulin (CBG), leaving ~5% of cortisol in the free form (84). CBG is primarily synthesized by the liver and secreted into the bloodstream, where it binds and provides a pool of circulating cortisol. It enhances the availability of cortisol to be released on demand both systemically and at a tissue level. Whereas plasma cortisol reflects total cortisol (i.e., bound and unbound), the salivary levels only reflect unbound/free cortisol. Changes in circulating CBG have a significant impact on total, but not free, cortisol concentrations (85, 86). The CBG is under a tight regulation by estradiol, with women expressing greater CBG basal concentrations than men (87). Interestingly, ethinyl estradiol, found in oral contraceptives (OC), dose-dependently increases CBG serum levels (88). Ethynyl estradiol is also a potent ER modulator, with 194 and 151% of the affinity of estradiol for the ER-α and ER-β, respectively (89). It is, however, unclear whether changes in estradiol across the menstrual cycles can alter CBG. Nenke et al. (90) report that total CBG concentration reaches ~1,000 nmol/L in pregnancy as well as during the active phase of pill cycle containing ethinyl estradiol. These expression levels are substantially higher than the CBG levels of ~500 nmol/L determined in non-pregnant, non-OC taking individuals (90). Unfortunately, this study did not account for menstrual cycle phase, which would have provided valuable information regarding CBG changes, if any, across the menstrual cycle. The question of potential CBG changes across the menstrual cycle due to changes in estradiol concentrations should be evaluated in future studies by carefully examining the potential anti-estrogenic and blunting effect of luteal progesterone on estradiol-induced increase in CBG (91).

There are several limitations to consider in the present meta-analysis. First, our sub-analyses may have been underpowered to detect effects of time variations. For example, the “time of day” sub-analysis showed a significant effect of morning, but not afternoon menstrual phase cortisol (Table 3). However, degrees of freedom for morning vs. afternoon sub-analyses were 21 and 7, respectively, with the afternoon sample possibly underpowered to detect a significant effect. Importantly, our sub-analysis of “source” (plasma vs. serum) was consistent with our main study results, showing higher follicular cortisol in both the free and total form. Furthermore, given that the overall sample was fairly homogenous, with most women in the same age range (20–29 years old) and a normal BMI (Table 1), we were unable to perform a meta-regression, which would have provided meaningful information related to the direction of future mechanistic studies.

As the research evaluating interacting effects between HPA and HPG axes unfolds, there are several issues to consider. Perhaps the greatest pitfall of menstrual cycle research is the inadequate assessment of the menstrual cycle phase. In the full-article evaluation step of our meta-analysis, we excluded findings from 44 studies because menstrual cycle phase was estimated based on self-report. Retrospective reports of menstrual cycle “start” and “duration” are plagued by profound phase misinformation (92) and prospective measures confirming both ovulation and luteal phase status are essential. Menstrual cycle phase determination was also based on self-report in approximately half of the studies evaluating the HPA reactivity in the follicular vs. luteal phase (16, 18, 19), contributing to the inability to make meaningful conclusions regarding the direction of effect.

HPA axis dysfunction is strongly implicated in the etiopathology of affective disorders (93–95) with women at an increased risk (96, 97). Yet, basic questions related to the function of the HPA axis throughout stages of the menstrual cycle under acute or prolonged stress conditions remain largely unanswered. Whereas, our meta-analysis reflects a single time point in the cortisol diurnal cycle, it is still unknown whether there are phase-specific effects on the shape of the diurnal curve. This assessment could be easily implemented given the availability of the salivary (unbound) cortisol assay, and would provide a comprehensive picture of daily cortisol trajectory and its potential mean, amplitude and/or phase shift as the hormonal milieu changes across the menstrual cycle. In this case, the awakening and morning cortisol should be taken more frequently than the late afternoon/evening samples.

It is strongly recommended that future studies employ a fine-grained approach (i.e., evaluating early/mid follicular, ovulatory, early, mid and late luteal phases) to advance women's mental health research, rather than broadly defining phases as “follicular” vs. “luteal.”

In summary, the aim of our meta-analysis was to summarize findings of a period spanning over 50 years of research and test whether circulating cortisol levels change as a function of menstrual phase. With respect to this objective, we showed higher cortisol levels in the follicular *vs*. luteal phase of the menstrual cycle. By completing this aim, our hope is to simultaneously increase awareness of the poor state of women's neuroendocrine science research. Experimental protocols designed to study effects that influence the HPA and HGA axis function need to be specifically designed to account for women's physiological requirements. The joint protocols previously designed for both sexes rarely apply to studies involving women. Whereas, participant recruitment, evaluation and analysis are more rapid in many research scenarios involving men, given the dynamic nature of the menstrual cycle and the need for prospective data collection, the same parameters should not be applied to research involving women. Resources and completion expectations need to be adjusted as such. Once implemented, these changes will contribute meaningful information to progress our understanding of rhythmic hormonal changes, which are crucial for understanding the now well-established sex difference in affective disorder development and progression.

## Data Availability Statement

The datasets generated for this study are available on request to the corresponding author.

## Author Contributions

AH, KK, SC, and TE-M conceptualized the question, completed literature search and data gathering. AH and FS completed statistical analysis. AH and GP wrote the manuscript.

## Conflict of Interest

The authors declare that the research was conducted in the absence of any commercial or financial relationships that could be construed as a potential conflict of interest.
