# Supplementary material for: Higher Circulating Cortisol in the Follicular vs. Luteal Phase of the Menstrual Cycle: A Meta-Analysis
Source: Front Endocrinol (Lausanne). 2020 Jun 2;11:311. doi: 10.3389/fendo.2020.00311 (PMC7280552; doi:10.3389/fendo.2020.00311)
Supplement: Supplementary file 1 [file Table_1.DOCX]

| Supplementary Table 1. Menstrual cycle and outcome measure information from individual studies | | | | | | |
| --- | --- | --- | --- | --- | --- | --- |
| **AUTHOR** | **YEAR** | **MENSTRUAL CYCLE** | | | **CORTISOL** | |
|  |  | **Follicular Phase** | **Luteal Phase** | **Phase Estimation** | **Time** | **Source** |
| Andreano | 2008 | 1 to 7 | 18 to 24 | Estradiol and progesterone | Afternoon | Saliva |
| Barbarino | 1989 | 4 to 8 | 20 to 24 | Estradiol and progesterone | Morning | Plasma |
| Beck | 1972 | 10 (II cycles) | 24 (II cycles) | LH surge | Morning and afternoon (plasma), cortisol secretion rate and urinary cortisol (24-hour) | Plasma and urine |
| Bement | 2009 | "Mid-follicular" | "Mid-luteal" | LH surge | Afternoon | Saliva |
| Bricout | 2003 | "Mid-follicular" | "Mid-luteal" | Estradiol and progesterone | 24-hour | Urine |
| Cannon | 1998 | 1 to 14 | 14 to 28 | Progesterone | 24-hour | Urine |
| Carr | 1979 | Values available for every day of the phase | Values available for every day of the phase | LH surge | Morning | Plasma |
| Caufriez | 2018 | 3 to 8 | 23 to 28 | Basal body temperature | 24-hour | Urine |
| Childs | 2010 | 3 to 10 | 16 to 24 | LH ovulation test | Morning | Plasma and Saliva |
| Collins | 1985 | 5 to 7 (II cycles) | 22 to 25 (II cycles) | Basal body temperature | Morning | Plasma |
| Espin | 2013 | 5 to 8 | 20 to 24 | Basal body temperature | Afternoon | Saliva |
| Genazzani | 1975 | Values available for every day of the phase | Values available for every day of the phase | LH surge | Morning | Plasma |
| Heitkemper | 1996 | 1, 2, 5, 6, 7 | 15, 19, 22, 25 | LH ovulation test | Morning and Afternoon | Urine |
| Huang | 2015 | 1 to 4 | 24 to 28 | Estradiol and Progesterone | Afternoon | Saliva |
| Inoue | 2007 | 1 to 14 | 14 to 21, 21 to 28 | Estradiol and Progesterone | Morning | Plasma |
| Judd | 1995 | 3 to 5 | 20-24 | LH ovulation test | 10-hour | Serum |
| Kasa-Vubu | 2005 | 1 to 14 | 14 to 28 | LH and Progesterone | 24-hour | Plasma |
| Kerdelhue | 2001 | Values available for every day of the phase | Values available for every day of the phase | LH surge | Morning and Afternoon | Serum |
| Kirschbaum | 1999 | 4 to 7 | 21 to 25 | Estradiol and progesterone | Afternoon | Plasma and Saliva |
| LeRoux | 2014 | 8 to 10 | 20 to 22 | Estradiol and progesterone | Morning, CAR and Afternoon | Saliva |
| Liu | 1987 | 1 to 5 | 20 to 22 | Pelvic Ultrasound | Morning | Plasma |
| Lombardi | 2004 | 5 to 7 | 22 to 26 | LH surge and progesterone | Morning | Serum |
| Maki | 2015 | 2 to 4 | 22 to 24 | LH ovulation test | Afternoon | Saliva |
| Ohara | 2015 | 1 to 14 | 14 to 28 | LH ovulation test | Morning | Saliva |
| Paoletti | 2006 | 5 to 8 | 21 to 24 | Basal body temperature | Morning | Serum |
| Parry | 2000 | 6 to 8 | 26 to 28 | LH ovulation test | Morning | Plasma |
| Rasgon | 2000 | 2 to 9 | 7 to 14 | LH ovulation test | Morning | Plasma |
| Reynolds | 2018 | 7 to 10 | 20 to 23 | LH ovulation test | Afternoon | Saliva |
| Roche | 2015 | 1 to 14 | 14 to 28 | Progesterone and Estradiol | Morning | Plasma and Saliva |
| Stewart | 1993 | 7 | 21 | Progesterone | 12-hour | Plasma |
| Su | 1997 | 3 to 7 days after the end of menses | 21 | Progesterone | Morning | Plasma |
| Timon | 2013 | 1 to 2 | 21 to 22 | Basal body temperature | Morning | Urine |
| Tulenheimo | 1987 | 6 to 9 | 21 to 24 | Progesterone | Morning | Plasma |
| Villada | 2017 | 5 to 8 | 20 to 24 | Basal body temperature | Afternoon | Saliva |
| Wolfram | 2011 | 2 to 6 | 21 to 24 | LH ovulation test | CAR | Saliva |
